# Revolutionary breakthrough: FDA approves Vyjuvek, the first topical gene therapy for dystrophic epidermolysis bullosa

**DOI:** 10.1097/MS9.0000000000001422

**Published:** 2023-10-17

**Authors:** Afsheen Khan, Rumaisa Riaz, Saad Ashraf, Aymar Akilimali

**Affiliations:** a Department of Internal Medicine, Dow University of Health Sciences, Karachi, Pakistan; b Faculty of Medicine, Official University of Bukavu, Bukavu, Democratic Republic of the Congo

**Keywords:** blister, collagen type VII, epidermolysis bullosa dystrophica, genetic therapy, humans, wound healing

## Abstract

This article provides an updated overview of Vyjuvek, a Food and Drug Administration (FDA) approved medication and its potential in managing dystrophic epidermolysis bullosa (DEB). DEB is a rare genetic disorder characterized by skin fragility, blistering, wounds, and scarring. The underlying cause of DEB is the impaired production of type VII collagen (COL7), leading to weakened anchoring fibrils in the skin. Vyjuvek is the first topical gene therapy for DEB, utilizing a genetically modified HSV-1 (herpes simplex virus 1) vector to express human COL7 and promote wound healing. Clinical trials have shown that Vyjuvek increases the probability of complete wound healing compared to placebo. Although further research is needed, Vyjuvek represents a significant advancement in addressing the unmet medical needs of patients with DEB, offering hope for improved quality of life and long-term complication reduction.

## Background

Dystrophic epidermolysis bullosa (DEB) is a rare inherited disorder characterized by skin fragility leading to blistering, wounds, and scarring^1^. DEB affects both males and females equally, with an estimated prevalence of two to six cases per million live births^2^. The article focuses on Vyjuvek, an FDA (Food and Drug Administration)-approved medication, exploring its characteristics, dosage, mechanism of action, and potential benefits in the management of DEB. It aims to provide valuable insights into this treatment option, helping people better understand the potential benefits of Vyjuvek in managing this rare inherited disorder.

## Pathophysiology

DEB is a disorder characterized by genetic variation that affects the adhesion components responsible for cell–cell or cell–matrix interactions. It can manifest as both autosomal dominant and recessive inheritance patterns^3^. The primary cause of DEB is the impaired production of type VII collagen (COL7). COL7 is a crucial protein for maintaining the structural integrity of the skin, present in the basement membrane of the dermis. Mutations in the COL7A1 (collagen type VII alpha 1 gene) gene, which encodes COL7, result in weakened anchoring fibrils, rendering the skin extremely fragile and prone to blistering, even with minor mechanical pressure^4^. The underlying pathophysiology involves a series of events, starting with faulty anchoring, followed by blister formation caused by friction, delayed wound healing, and progression of chronic ulcers, fibrosis, and scarring^5^.

## Current treatment/adjunct therapy

### Whirlpool therapy as an adjunct for treating DEB

Whirlpool therapy, also known as hydrotherapy, is a helpful adjunct available in most hospitals and assists in the care of inpatients with epidermolysis bullosa. This technique uses warm water and special tubs to create a massaging action to achieve improved therapeutic blood flow to the area. The warm water and whirlpool cause blood vessels to dilate, thereby increasing blood circulation. The warm water and whirlpool expand blood vessels, enhancing blood circulation and wound healing and consequently relieving disorders such DEB^6^.

### Vyjuvek (Beremagene-geperpavec): first topical gene therapy

There is no specific treatment for DEB and symptomatic management remains the cornerstone of care^2^. To provide patients with the best care, it is essential to avoid new blisters and trauma, and to properly dress wounds^2^. However, the FDA’s approval of Vyjuvek (Beremagene-geperpavec), the first gene therapy medication for DEB to get approval, is a significant step forward in addressing the unmet medical needs of DEB patients^7^. The herpes simplex virus 1 (HSV-1) vector-based gene therapy suspension Vyjuvek (Beremagene geperpavec-svdt) was combined with a sterile excipient gel for topical application to the wounds. The human COL7 protein is expressed using this genetically modified live replication-defective HSV-1-based vector^8^.

Unlike conventional treatments, which focus on symptom management and supportive care, Vyjuvek seeks to address the core cause of the disease by identifying and correcting the genetic abnormalities that cause DEB. This innovative therapy initiates the transcription of encoded human COL7A1 in the nucleus, restoring functional protein COL7 in the cell while also boosting keratinocyte and fibroblast development. As a result, the wound-healing process is accelerated^9^. These COL7 molecules group together to create long, slender bundles known as anchoring fibrils. Anchoring fibrils are crucial for preserving the integrity of the skin because they hold the epidermis and dermis together. In individuals with DEB, topical application of Beremagene-geperpavec (B-VEC) topically showed a higher probability of achieving complete wound healing at 3 and 6 months compared to the use of a placebo^10^. The utilization of a solitary intradermal injection of allogeneic fibroblasts in patients afflicted with recessive DEB (RDEB) induces transient amplification at the pace of recovery^11^. In contrast to this, Vyjuvek may result in one time treatment with long-term effects and patients may require fewer hospital visits and medical interventions which makes it distinct from existing treatments available. Furthermore, this technique may be able to prevent or reduce the occurrence of DEB-related issues like persistent wounds, infections, and discomfort, which are typically observed in standard treatments^11^.

## Clinical trial

The safety and efficacy of Vyjuvek were principally demonstrated in a randomized, double-blinded, placebo-controlled study including 31 patients (20 males and 11 females), with 30 patients having autosomal recessive DEB and one with autosomal dominant DEB. Two DEB wounds of roughly the same size on each patient were chosen for the trial, and they were randomly assigned to receive either a placebo (excipient gel) or a weekly topical treatment of Vyjuvek for 26 weeks. The difference between the proportion of full (100%) wound closure at 24 weeks served as the foundation for determining the efficacy. Only 26% of the wounds receiving a placebo treatment were completely closed, compared to 65% of the wounds receiving Vyjuvek treatment^7,12^.

Additionally, the safety profile of Vyjuvek was assessed in a phase 1/2 study involving 12 individuals, all of whom were at least 6 years old and had been previously diagnosed with generalized RDEB. In this trial, one wound was subjected to treatment with Vyjuvek, a therapeutic agent, while another wound received a placebo. The trial successfully achieved its primary and secondary objectives, which included assessing C7 expression, anchoring fibril assembly, reduction in wound surface area, duration of wound closure, and time taken for wound closure following treatment with B-VECs^12^. The above two clinical trials are reported in Table 1 with their study population, outcomes, and adverse effects^13^. The above two clinical trials are reported in Table 1 with their study population, outcomes, and adverse effects.

**Table 1:** summary of clinical trials.

Study ID	Drug	Phase	Sample size	Outcomes	Adverse effect
GEM-1 trial (NCT03536143)	One wound was treated with Vyjuvek, while a different wound was administered with a placebo	Phase 1/2	12 people at least 6 years old and had been diagnosed with generalized RDEB	Primary and secondary objectives of C7 expression, anchoring fibril assembly, wound surface area reduction, duration of wound closure, and time to wound closure following B-VEC treatment were met	No deaths, serious or significant adverse events were reported
GEM-3 study (NCT04491604)	One wound was treated with Vyjuvek, while another wound was administered the placebo	Phase 3	31 people with DEB, ages 1–44	The primary endpoint was complete wound healing of treated as compared with untreated wounds at 6 months. Secondary endpoints included complete wound healing at 3 months and the change from baseline to weeks 22, 24, and 26 in pain severity	The most common adverse events were pruritus, chills, and squamous-cell carcinoma of the skin, each of which occurred in 3 patients (10%)

B-VEC: Beremagene-geperpavec; RDEB, recessive dystrophic epidermolysis bullosa.

## Recommended dosage, side effects, and precautions

Vyjuvek gel is administered topically to the wound(s) once a week at a dosage based on age. The maximum weekly dose and volume for children aged 6 months to 3 years old are, respectively, 1.6×10^9^ PFU (plaque-forming unit) and 0.8 ml. Vyjuvek is a biological suspension that ranges in color from opalescent yellow to colorless. The excipient gel is a clear, viscous solution that is given in a 1.5 ml fill volume in a unique, blue-capped vial. Prior to administration, the Vyjuvek biological suspension (1.0 ml) was combined in an excipient gel vial^7^.

Vyjuvek may lead to certain adverse reactions, with the most commonly reported being itching, chills, redness, rash, cough, and runny nose. It is important to note that these adverse reactions are the most frequently observed, but may not occur in every individual using Vyjuvek. If any of these symptoms persist or worsen, it is advisable to consult a healthcare professional for further guidance and evaluation^8^.

Vyjuvek, a non-replicative substance, does not integrate with the genetic material of the subject’s cells. Clinicians should be mindful of the following precautions to ensure safety during its application. First, it is essential to avoid direct contact with the wounds and their dressings for around 24 h following treatment, making sure not to touch or scratch them during this period. Secondly, when providing assistance to individuals in changing wound dressings or handling the disposal of dressings, it is crucial to wear protective gloves at all times. Finally, in the unfortunate event of accidental exposure, such as a splash to the eyes or mucous membranes, immediate action is required. The affected area should be thoroughly rinsed with clean water for a minimum of 15 min. Being vigilant about these precautions will help ensure the safety and well-being of both the clinicians and the patients under their care^7^.

## Conclusion

Although DEB remains a challenging condition to manage, it has emerged as an FDA-approved drug with promising characteristics, dosage, and mechanisms of action. Further research and clinical trials are necessary to fully understand its efficacy and safety profile. However, Vyjuvek offers hope for individuals with DEB, potentially providing relief from blistering, promoting wound healing, and reducing long-term complications of the disease. Continued advancements in the development of targeted therapies, such as Vyjuvek, hold great potential for improving the quality of life of individuals living with DEB.

## Ethical approval

The paper did not involve patients; therefore, no ethical approval was required.

## Consent

The study was not done on patients or volunteers; therefore, no written consent was required.

## Sources of funding

No funding was acquired for this paper.

## Author contribution

A.K.: conceptualization, writing – original draft, final approval, and agreeing to the accuracy of the work; R.R. and S.A.: writing – original draft, final approval, and agreeing to the accuracy of the work; A.A.: reviewing original draft, final approval, and agreeing to the accuracy of the work.

## Conflicts of interest disclosure

There are no conflicts of interest.

## Research registration unique identifying number (UIN)

1. Name of the registry: not applicable.
2. Unique identifying number or registration ID: not applicable.
3. Hyperlink to your specific registration (must be publicly accessible and will be checked): not applicable.

## Guarantor

Afsheen Khan and Aymar Akilimali.

## Data availability statement

Not applicable.

## Provenance and peer review

Not commissioned, externally peer-reviewed.

## References

[1] Intong LRA Murrell DF. Inherited epidermolysis bullosa: new diagnostic criteria and classification. Clin Dermatol 2012;30:70–77. 22137229 10.1016/j.clindermatol.2011.03.012

[2] Shinkuma S. Dystrophic epidermolysis bullosa: a review. Clin Cosmet Investig Dermatol 2015;8:275. 10.2147/CCID.S54681 PMC4451851 26064063

[3] Fine JD Bruckner-Tuderman L Eady RAJ. Inherited epidermolysis bullosa: updated recommendations on diagnosis and classification. J Am Acad Dermatol 2014;70:1103–1126. 24690439 10.1016/j.jaad.2014.01.903

[4] Dang N Murrell DF. Mutation analysis and characterization of COL7A1 mutations in dystrophic epidermolysis bullosa. Exp Dermatol 2008;17:553–568. 18558993 10.1111/j.1600-0625.2008.00723.x

[5] Chung HJ Uitto J. Type VII collagen: the anchoring fibril protein at fault in dystrophic epidermolysis bullosa. Dermatol Clin 2010;28:93–105. 19945621 10.1016/j.det.2009.10.011 PMC2791403

[6] Epidermolysis Bullosa Treatment & Management: Medical Care, Surgical Care, Consultations. Accessed 13 September 2023. https://emedicine.medscape.com/article/1062939-treatment.

[7] FDA, Cber. Highlights of Prescribing Information Full Prescribing Information: Contents* 1 Indications and Usage 2 Dosage and Administration 2.1 Dose 2.2 Preparation 2.3 Administration 3 Dosage Forms and Strengths 4 Contraindications 5 Warnings and Precautions 5. Accessed 13 September 2023. http://www.fda.gov/medwatch

[8] Vyjuvek | FDA. Accessed 13 September 2023. https://www.fda.gov/vaccines-blood-biologics/vyjuvek

[9] Dhillon S. Beremagene geperpavec: first approval. Drugs 2023;83:1131–1135. 37432558 10.1007/s40265-023-01921-5

[10] Guide SV Gonzalez ME Bağci IS. Trial of beremagene geperpavec (B-VEC) for dystrophic epidermolysis bullosa. N Engl J Med 2022;387:2211–2219. 36516090 10.1056/NEJMoa2206663

[11] Petrof G Martinez-Queipo M Mellerio JE. Fibroblast cell therapy enhances initial healing in recessive dystrophic epidermolysis bullosa wounds: results of a randomized, vehicle‐controlled trial. Br J Dermatol 2013;169:1025–1033. 24032424 10.1111/bjd.12599

[12] Study Record | ClinicalTrials.gov. Accessed 13 September 2023. https://clinicaltrials.gov/study/NCT04491604?tab=results

[13] Gurevich I Agarwal P Zhang P. In vivo topical gene therapy for recessive dystrophic epidermolysis bullosa: a phase 1 and 2 trial. Nat Med 2022;28:780–788. 35347281 10.1038/s41591-022-01737-y PMC9018416

